# Shelby Medical College

**Published:** 1858-09

**Authors:** 


Shelby Medical College.—It will be perceived by our advertising sheet that the Faculty of this School is now fully organized. The name of Dr. Atchison, mentioned in our last number, does not appear in the announcement. Dr. Wright, late of the Memphis Medical College, occupies the Chair of Physiology and Pathology, and Dr. Maddin, of Nashville, that of Anatomy. The latter gentleman is a graduate of the University of Louisville, in the time of Drake, Caldwell, Short, Cobb, and Miller, and is said to be a superior lecturer.

